# Molecular basis for Eaf3-mediated assembly of Rpd3S and NuA4

**DOI:** 10.1038/s41421-023-00565-9

**Published:** 2023-05-26

**Authors:** Zhenzhen Chen, Taylor Lundy, Zhongliang Zhu, Victoria E. Hoskins, Jiahai Zhang, Xuebiao Yao, Brian D. Strahl, Chao Xu

**Affiliations:** 1 The First Affiliated Hospital of University of Science and Technology of China, Hefei, Anhui China; 2 MOE Key Laboratory for Cellular Dynamics, University of Science and Technology of China, Hefei, Anhui China; 3 Department of Biochemistry and Biophysics, School of Medicine, University of North Carolina at Chapel Hill, Chapel Hill, NC USA

**Keywords:** X-ray crystallography, Epigenetics

Dear Editor,

In *Saccharomyces cerevisiae*, reduced potassium dependency-3 (Rpd3) resides in two distinct histone deacetylase complexes, termed Rpd3S and Rpd3L^1–3^. The Rpd3S complex functions to repress transcriptional initiation within the coding region of genes during RNA polymerase II elongation by maintaining hypoacetylation^2,4^. Eaf3 and Rco1 are unique members of Rpd3S. Eaf3 binds to di- and tri-methylation of histone H3 at lysine 36 (H3K36me2/3) via its N-terminal chromo barrel domain (Eaf3^CBD^)^5^, and it also resides in the NuA4 histone acetyltransferase complex^4–6^. Rco1 recognizes unmodified H3K4 (H3K4me0) via its PHD1 domain and associates with Eaf3 to enhance the Eaf3–H3K36me3 interaction^7^. Thus, H3K4me0 and H3K36me recognition by Rpd3S enforces the targeting and activity of Rpd3S within gene bodies that are rich in H3K36 methylation but depleted in H3K4 methylation^8^. However, the underlying molecular mechanisms of Eaf3–Rco1 interaction are largely unknown.

First, we purified the C-terminal MRG domain of Eaf3 (Eaf3^MRG^, aa 218–401) (Fig. 1a), and confirmed its binding to the Rco1^PHD1–SID^ (aa 240–376) by GST pull-down assay (Supplementary Fig. S1a). Then by using the isothermal titration calorimetry (ITC) binding assay, we found that the H3K4me0, H3K4me1, and H3K4me2 peptides bind to the Eaf3^MRG^–Rco1^PHD1–SID^ complex with *K*_D_ values of 22 μM, 65 μM, and 360 μM, respectively, whereas H3K4me3 abolishes the binding (Fig. 1b).

**Fig. 1:**
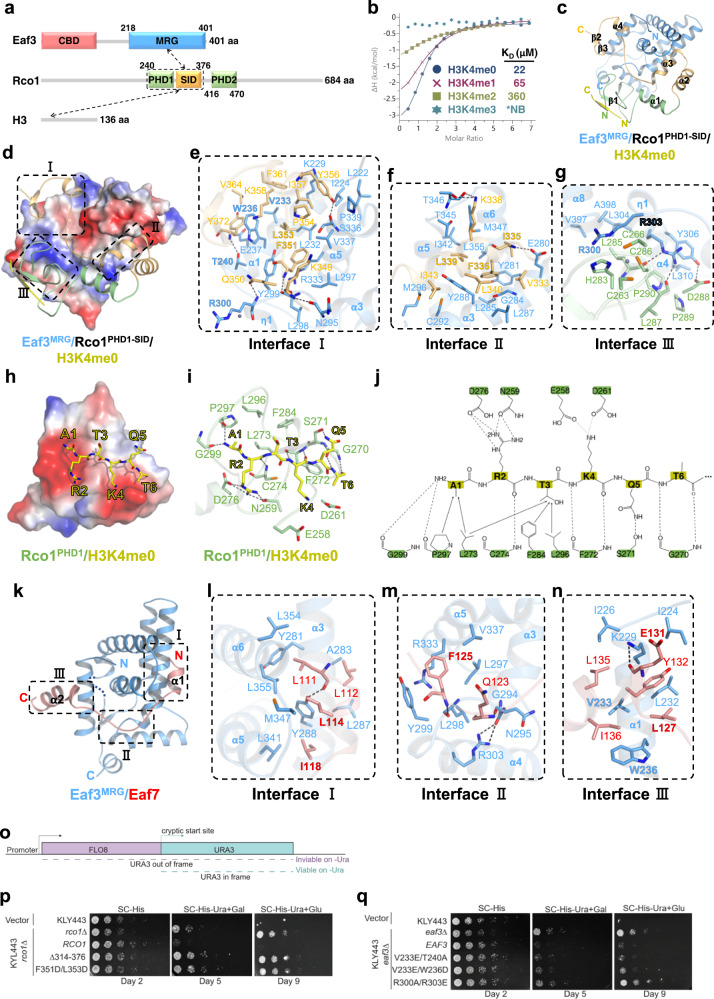
Structures of Eaf3 complexes. **a** Domain architecture of Eaf3 and Rco1 with the intermolecular interactions indicated by arrows. The boundaries of Eaf3^MRG^, Rco1^PHD1–SID^, and Rco1^PHD2^ are labeled. **b** ITC binding curves for Eaf3^MRG^/Rco1^PHD1–SID^ with H3K4me0^1–12^, H3K4me1^1–12^, H3K4me2^1–12^, and H3K4me3^1–12^. **c** Overall structure of Eaf3^MRG^–Rco1^PHD1–SID^ bound with H3K4me0. Eaf3^MRG^, Rco1^PHD1^, Rco1^SID^, and H3K4me0 are shown in blue, green, orange, and yellow cartoons, respectively. Secondary structures of Rco1^PHD1–SID^ are labeled. **d** The electrostatic surface of Eaf3^MRG^ bound with Rco1^PHD1–SID^, which is shown in the same way as in Fig. 1c. **e**–**g** Close-up interactions between Eaf3^MRG^ and the C-terminal region (**e**), the α3 (**f**), and the N-terminal region (**g**) of Rco1^SID^. The interface residues that are used in mutagenesis and pull-down binding experiments are highlighted. The Zn ion is shown as a gray sphere. **h** The electrostatic surface of Rco1^PHD1^ bound with H3K4me0 (yellow sticks). **i** Close-up interaction of the Rco1^PHD1^/H3K4me0 interface. **j** Interaction diagram between Rco1^PHD1^ and the H3K4me0 peptide. **k** Overall structure of Eaf3^MRG^ (blue) bound to Eaf7^108–143^ (red). **l**–**n** Close-up interaction between Eaf3^MRG^ and the α1 of Eaf7 (**l**), the linker of Eaf7 (**m**), and the α2 of Eaf7 (**n**). **o** Overview of the cryptic transcription reporter system. **p**, **q** Spotting assays of the strains used in this study on synthetic complete (SC) agar lacking histidine (**–**His) and uracil (**–**Ura) with either glucose (Glu) or galactose (Gal). Shown are 2-fold or 5-fold serial dilutions and the indicated days.

To gain mechanistic insights into the Eaf3–Rco1 interaction and the preference of Rco1 for H3K4me0, we solved the complex structure of Eaf3^MRG^–Rco1^PHD1–SID^–H3K4me0^1–12^ at 1.60 Å resolution (Fig. 1c; Supplementary Table S1). In the ternary complex, Eaf3^MRG^ adopts a canonical fold similar to that of MRG15^MRG^ (Supplementary Figs. S2a–c, S3a), and buries an area of approximately 2072 Å^2^ with Rco1^PHD1–SID^. Rco1^PHD1–SID^ interacts with Eaf3^MRG^ via three interfaces, with two for SID (interfaces I and II) and one for PHD1 (interface III) (Fig. 1d).

The Rco1^SID^ C-terminal region contacts α1, α3, and α5 of Eaf3^MRG^, with the loop between α3 and α4 interacting with Eaf3^MRG^ via hydrophobic and hydrogen bonding interactions. Phe351, Leu353, and Pro354 of Rco1 form hydrophobic interactions with Leu232, Val233, Trp236, Tyr299, Arg333, and Val337 of Eaf3; Lys349 and Phe351 of Rco1 form two main chain hydrogen bonds with Asn295 and Leu298 of Eaf3, respectively; the Rco1 Gln350 forms a hydrogen bond with Eaf3 Arg300 (Fig. 1e). α4, β2, and β3 of Rco1^SID^ interact with Eaf3^MRG^ mainly via hydrophobic interactions. Tyr356, Ile357, and Lys358 of Rco1^SID^ form hydrophobic interactions with Leu222, Ile224, Lys229, Leu232, Trp236, and Pro339 of Eaf3; Phe361, Val364, and Tyr372 of Rco1 also form hydrophobic interactions with Lys229, Val233, and Trp236 of Eaf3; the main chains of Rco1 Tyr356 and Tyr372 are hydrogen-bonded to Eaf3 Lys229 and Thr240, respectively. The Rco1 Tyr356 forms one additional hydrogen bond with Eaf3 Ser336 (Fig. 1e).

α3 of Rco1^SID^ further makes hydrophobic interactions with α3, α5, and α6 of Eaf3^MRG^. Ile335, Phe336, and Leu339 are accommodated into a large hydrophobic concave of Eaf3^MRG^ formed by Tyr281, Gly284, Leu285, Ile342, Thr345, Met347, and Leu355; Val333, Leu340, and Ile343 also make hydrophobic contacts with the residues in Eaf3^MRG^ α3, including Gly284, Leu287, Tyr288, Cys292, and Met296. In addition to hydrophobic interactions, the main chain amide group of Rco1 Ile335 is hydrogen-bonded to the side chain carboxyl group of Eaf3 Glu280, and Lys338 of Rco1 also forms two hydrogen bonds with Eaf3 Thr346 (Fig. 1f).

Unexpectedly, Rco1^PHD1^ also interacts with α3, η1, α4, and α8 of Eaf3^MRG^ via electrostatic and hydrophobic interactions, albeit fewer than those found between Rco1^SID^ and Eaf3^MRG^. The main chain carbonyl groups of Rco1 Leu285 and Leu287 are hydrogen bonded to the side chain of Eaf3 Arg303; Rco1 Asp288 forms one hydrogen bond with the side chain of Eaf3 Tyr306; Rco1 Leu285 forms hydrophobic interactions with Leu304, Val397, and Ala398 of Eaf3; Pro289 and Pro290 of Rco1 make additional hydrophobic contacts with Tyr306 and Leu310 of Eaf3; and the imidazole ring of Rco1 His283 further stacks with the side chain of Eaf3 Arg300 (Fig. 1g). The interactions between Eaf3^MRG^ and Rco1^PHD1^ raise the possibility that Eaf3^MRG^ allosterically reinforces the Rco1–H3 interaction by stabilizing Rco1^PHD1^. Consistently, ITC binding assays demonstrated that the Eaf3–Rco1 complex binds to the H3K4me0 peptide with 5-fold higher affinity than Rco1^PHD1^ alone (*K*_D_ values: 22 μM vs 110 μM) (Fig. 1b; Supplementary Fig. S4a), underscoring the Eaf3^MRG^–Rco1^PHD1^ interaction mediated by Arg300 and Arg303 of Eaf3^MRG^ (Supplementary Fig. S4b).

We next employed structure-guided mutagenesis and affinity pull-down assays to evaluate the roles of Eaf3 and Rco1 residues in complex formation. Consistent with the structural analysis, V233E/T240A, V233E/W236D, and R300A/R303E of Eaf3^MRG^ impair the binding to Rco1^PHD1–SID^ (Supplementary Fig. S1a, b), and a truncated form of Rco1^PHD1–SID^ (Rco1^240–313^) displays no binding towards Eaf3^MRG^ (Supplementary Fig. S1c, d). While I335A/F336A/L339A of Rco1^PHD1–SID^ does not disturb the binding to Eaf3^MRG^, an F351D/L353D mutation in Rco1^SID^ does (Supplementary Fig. S1c, d). Together, these assays further validate the Eaf3^MRG^–Rco1^PHD1–SID^ interface.

Similar to previously reported PHD complex structures^9^, the H3K4me0 peptide lies in the negative surface of Rco1^PHD1^ by forming a two-stranded antiparallel β-sheet with Rco1^PHD1^ β1 (Fig. 1h). H3A1 makes hydrophobic interactions with Leu273 and Pro297, and forms two main chain hydrogen bonds with Pro297 and Gly299 of Rco1; the side chain of H3R2 forms hydrogen bonds with Asn259 and Asp276 of Rco1; H3T3 forms hydrophobic interactions with Leu273, Phe284, and Leu296 of Rco1 (Fig. 1i, j); the H3K4 side chain makes favorable electrostatic interactions with the side chains of Glu258 and Asp261 (Fig. 1b, i, j); H3Q5 also forms one side chain hydrogen bond with Ser271 (Fig. 1i, j). Collectively, the first five residues of the H3K4me0 peptide are specifically recognized by Rco1^PHD1^ (Fig. 1i, j; Supplementary Fig. S3b).

In addition to its role in Rpd3S, Eaf3 also forms a subcomplex with Eaf5 and Eaf7 in the NuA4 complex^10^. However, the mechanism underlying the interaction of Eaf3 with Eaf5 or Eaf7 remains elusive. To address these unknowns, we identified a region within Eaf7 (aa 108–143) as the Eaf3^MRG^ binding motif using GST pull-down assays (Supplementary Fig. S5a, b) and solved the structure of Eaf3^MRG^ bound with Eaf7^108–143^ at a resolution of 2.40 Å (Supplementary Table S1). Residues 111–142 of Eaf7 are visible in the complex and adopt two helices (α1 and α2) connected by a long linker (Fig. 1k; Supplementary Fig. S3c).

Eaf7^108–143^ binds to Eaf3^MRG^ mainly via hydrophobic interactions. α1 of Eaf7^108–143^ interacts with α3, α5 and α6 of Eaf3^MRG^ mainly via hydrophobic interactions (Fig. 1l); the linker region of Eaf7 interacts with α3, α4, and α5 of Eaf3^MRG^ via hydrophobic and hydrogen bonding interactions (Fig. 1m); α2 of Eaf7^108–143^ packs against α1 of Eaf3^MRG^. The main chain carbonyl group of Eaf7 Glu131 is hydrogen-bonded to Eaf3 Lys229, which forms an additional polar interaction with the side chain of Glu131 (Fig. 1n).

Consistently, mutagenesis and GST pull-down assays showed that L114A, I118A, R121A, D124A, and E131A of Eaf7^108–143^ slightly reduce the binding to Eaf3^MRG^, while F125A and L127A of Eaf7^108–143^ abolish the binding (Supplementary Fig. S5a, b). In addition, R300A/R303E, V233E/W236D, and V233E/T240A of Eaf3^MRG^ disrupt the binding to Eaf7^108–143^ (Supplementary Fig. S5c, d), suggesting that Eaf7^108–143^ and Rco1^SID1–PHD^ bind to the same site on Eaf3^MRG^. Furthermore, ITC binding assay showed that SUMO–Eaf7^108–143^ binds to Eaf3^MRG^ with a *K*_D_ value of 1.0 μM (Supplementary Fig. S6a) but displays no binding toward the Eaf3^MRG^–Rco1^PHD1–SID^ complex (Supplementary Fig. S6b). As a control, the binding data showed that SUMO alone does not bind to Eaf3^MRG^ (Supplementary Fig. S6c). Taken together, the structural analysis and binding data confirm that Eaf7^108–143^ and Rco1^PHD1–SID^ bind to the same surface of Eaf3^MRG^.

Then, we compared the two Eaf3^MRG^ complexes with that of MRG15^MRG^/MRGBP (PDB: 2N1D) (Supplementary Fig. S7). All MRG-binding ligands contain a common helix–linker–helix motif, such as α3–α4 of Rco1, α1–α2 of Eaf7, and α2–α3 of MRGBP (Supplementary Fig. S7a–c). However, Rco1^SID^ contains an additional C-terminal β-sheet, and its α2–α3 deviates from the α1–α2 of MRGBP. Most importantly, Rco1^PHD1^ makes additional contact with Eaf3^MRG^ in the complex (Supplementary Fig. S7d).

An established cryptic reporter assay^11^ with a modified *FLO8* gene was employed to evaluate the biological significance of the Eaf3–Rco1 interaction (Fig. 1o). Consistent with structural analysis, strains depleted of *RCO1* resulted in cryptic transcription phenotypes that were rescued upon the expression of wild-type *RCO1* gene, but not the expression of its truncated form (∆314–376) or mutated form (F351D/L353D) (Fig. 1p). All Eaf3 mutants tested (V233E/T240A, V233E/W236D, and R300A/R303E) revealed varying degrees of cryptic transcription on galactose- or glucose-containing plates, with R300A/R303E exhibiting the strongest cryptic phenotype (Fig. 1q). As functional screens for cryptic transcription have not revealed a role of NuA4 in this process^12^, the cryptic phenotypes for our Eaf3 mutants most likely result from defects in Rpd3S. The expression of Eaf3 and Rco1 HA-tagged constructs was verified with G6PDH as the control (Supplementary Fig. S8a, b). Together, the in vivo assay highlights the importance of the Eaf3–Rco1 interaction in Rpd3S-mediated epigenetic regulation.

In this study, we presented the structures of Eaf3^MRG^ bound with Rco1^PHD1–SID^ and H3K4me0 or with a fragment of Eaf7. In the structure of Eaf3–Rco1–H3K4me0 complex, Eaf3^MRG^ allosterically enhances Rco1–H3K4me0 binding, implying that allosteric regulators might confer Eaf3^CBD^ the preference for H3K36me3 in the context of the Rpd3S complex. In addition, the solved structures confirm that Eaf7 and Rco1 bind to the same surface on Eaf3^MRG^, which complements our understanding of how Eaf3 resides in distinct histone modification complexes. Further work is required to delineate how the intricate regulatory role of Eaf3 is achieved through being incorporated exclusively into key chromatin-modifying complexes.

## Supplementary information

Supplementary Information

## Data Availability

Atomic coordinates and structure factors for the two Eaf3^MRG^ structures have been deposited into the Protein Data Bank under accession codes 8I3F and 8I3G.
