# Author Correction: Molecular basis for Eaf3-mediated assembly of Rpd3S and NuA4

**DOI:** 10.1038/s41421-023-00574-8

**Published:** 2023-06-30

**Authors:** Zhenzhen Chen, Taylor Lundy, Zhongliang Zhu, Victoria E. Hoskins, Jiahai Zhang, Xuebiao Yao, Brian D. Strahl, Chao Xu

**Affiliations:** 1 The First Affiliated Hospital of USTC, Division of Life Sciences and Medicine, University of Science and Technology of China, Hefei, Anhui China; 2 MOE Key Laboratory for Cellular Dynamics, University of Science and Technology of China, Hefei, Anhui China; 3 Department of Biochemistry and Biophysics, School of Medicine, University of North Carolina at Chapel Hill, Chapel Hill, NC USA

**Keywords:** X-ray crystallography, Epigenetics

Correction to: *Cell* *Discovery* (2023) 9:51

10.1038/s41421-023-00565-9 published online 26 May 2023

In this article, the authors have found an error in the affiliations. The first affiliation is incorrect. The correct one is “The First Affiliated Hospital of USTC, Division of Life Sciences and Medicine, University of Science and Technology of China, Hefei, Anhui, China.” We are sorry for the inconvenience.

