# Supplementary material for: Molecular basis for Eaf3-mediated assembly of Rpd3S and NuA4
Source: Cell Discov. 2023 May 26;9:51. doi: 10.1038/s41421-023-00565-9 (PMC10220053; doi:10.1038/s41421-023-00565-9)
Supplement: Supplementary file 1 — Supplementary Information [file 41421_2023_565_MOESM1_ESM.pdf]

Supplementary data S1

Protein expression and purification

Genes encoding Rco1$^{PHD1\text{-}SID}$ (residues 240-376), Eaf7$^{108\text{-}143}$, and Eaf3^{MRG} (residues 218-401) were amplified by polymerase chain reactions (PCR) from *S. cerevisiae* genome DNA. Eaf3^{MRG} was cloned into a modified pETDuet-1 (Novagen) with either Rco1$^{PHD1\text{-}SID}$ or Eaf7$^{108\text{-}143}$ with Eaf3^{MRG} at the upstream site and Rco1$^{PHD1\text{-}SID}$ or Eaf7$^{108\text{-}143}$ at the downstream site. For the proteins used for pulldown assay, the gene encoding Eaf3^{MRG} was cloned into modified pET28-SUMO vector; genes encoding Eaf3^{MRG}, Rco1$^{PHD1\text{-}SID}$, and truncated form of Rco1$^{PHD1\text{-}SID}$ (residues 240-376) were cloned into modified pET28-MBP vector. Genes encoding Eaf3^{MRG} and Eaf7$^{108\text{-}143}$ were cloned into pGEX-4T-1. The plasmids were transferred into the *Escherichia coli* BL21 (DE3), and the cells were grown in LB medium supplemented with 0.1 mg/mL ampicillin at 37 °C until OD_{600} reached 0.8. Recombinant proteins were expressed after induction at 16°C for 20 h by adding isopropyl-β-D-thiogalactopyranoside (IPTG) to a final concentration of 0.2 mM and ZnSO4 to a final concentration of 0.1 mM. Cells were harvested by centrifugation at 4,000 rpm for 15 min, and then were resuspended using binding buffer (20 mM Tris-HCl, pH 7.5, 400 mM NaCl) and were lysed by sonication on ice. Lysate was centrifuged at 10,000 rpm, 4 °C for 35 min and supernatant was collected for protein purification.

His-tagged, MBP-tagged, and SUMO-tagged proteins were purified with fast flow Ni-NTA column (GE healthcare) by washing with a buffer containing 20 mM Tris-HCl, pH 7.5, 400 mM NaCl, 20 mM imidazole and elution with a buffer containing 20 mM Tris-HCl, pH

7.5, 400 mM NaCl, 500 mM imidazole. The proteins were further purified by Gel filtration using HiLoadTM 16/ 600 superdexTM 75 pg column (GE healthcare) and ion exchange columns (HitrapTM Q HP, GE healthcare). The fractions corresponding to recombinant complexes were collected and concentrated to 20 mg/mL for further use. The mutants were constructed by conventional PCR using a MutanBEST kit (TaKaRa) and verified by DNA sequencing. The mutants were expressed and purified in the same way as the wild type proteins.

Crystallization, data collection and structure determination

All crystals were grown by using sitting drop vapor diffusion method at 18°C. For the $Eaf3^{MRG}$-$Eaf7^{108-143}$ complex, crystals were grown by mixing equal volume of protein and a buffer containing 0.1 M Tris-HCl, pH 8.5, 25% (w/v) PEG 4000, 0.025% (v/v) Dichloromethane. For crystallization of the $Eaf3^{MRG}$-$Rco1^{PHD1-SID}$ with $H3^{1-12}$, the $Eaf3^{MRG}$-$Rco1^{PHD1-SID}$ complex were pre-incubated with the synthesized unmethylated histone H3K4 peptide (GL Biochem) at a molar ratio of 1:3 at 4 °C for 30 min. The crystals were grown mixing equal volume of protein complex with a buffer containing 0.2 M $MgCl_2$, 0.1 M tris pH 8.5, 25% (w/v) PEG 4000, and 0.2 M NDSB-201. Before flash-freezing crystals in liquid nitrogen, all crystals were soaked in a cryo-protectant consisting of 90% reservoir solution plus 10% glycerol.

The diffraction data were collected on beam line BL18U1 at the Shanghai Synchrotron Facility (SSRF)[1]. All diffraction images were processed, integrated, and scaled using the HKL2000/3000 program [2,3]. The structures were solved by molecular replacement using Phaser, then the models were built manually by Coot [4] and refined by Phenix [5].

Glutathione S-transferase (GST) pull down

100 μg of purified GST-tagged proteins were added into and incubated with 100 μL of Glutathione Sepharose beads (GE Healthcare) at 4°C for 30 min in a buffer containing 20 mM Tris-HCl, pH 7.5, 200 mM NaCl. 150 μg of purified MBP-tagged or SUMO-tagged proteins were added and incubated at 4°C for 30 min. Beads were subsequently washed three times with 1 mL of washing buffer containing 20 mM Tris-HCl, pH 7.5, 200 mM NaCl, 1% glycerol, and 0.1% Triton X-100). The proteins bound with the Glutathione Sepharose beads were eluted by 50 μL buffer containing 20 mM Tris-HCl pH 7.5, 200 mM NaCl, 50 mM Glutahione, and examined by SDS-PAGE.

Isothermal titration calorimetry (ITC)

Peptides were synthesized by GL Biochem (Shanghai) Ltd. Before use, they were dissolved in a buffer containing 20 mM Tris-HCl, pH 7.5, 150 mM NaCl as a stock of 35-50 mM. Proteins were dialyzed against the same buffer. ITC experiments were performed by titrating 2 μL of peptide (2 mM) into cell containing 50 μM proteins on MicroCal PEAQ ITC (Malvern Panalytical, UK) at 25°C, with a spacing time of 120 s and a reference power of 5 μCal/s. Control experiments were performed by injecting the peptides into ITC buffer. Binding isotherms were plotted, analyzed and fitted in a one-site binding model with MicroCal PEAQ-ITC Analysis Software after subtraction of respective controls (Malvern Panalytical, UK).

Yeast strains, plasmids, materials, and instruments.

The cryptic reporter strain used in this study (KLY443), and a version that has been deleted for *RCO1* (KLY550) have been described previously [6,7]. KLY443 was used to generate a deletion of *EAF3* using standard gene deletion methods (yeast strains are listed in Supplementary Table

S2). The plasmids used for this study outlined in Supplementary Table S3. Primers used for polymerase chain reactions (PCR) to engineer the *eaf3*Δ and verify deletions were purchased from Integrated DNA Technologies (IDT) (Research Triangle Park, NC, USA) and are listed in Supplementary Table S3. Validation of plasmid sequences was conducted through Plasmidsaurus (Eugene, OR, USA). All other cloning reagents were purchased from New England BioLabs (NEB; Ipswich, MA, USA). DNA purification spin kits QIAquick Gel Extraction Kit and QIAprep Spin Miniprep Kit were purchased from Qiagen (Valencia, CA, USA) and used according to manufacture directions. All chemicals and buffer components, which were used without any further purification, were purchased from Sigma-Aldrich or VWR (Atlanta, GA, USA).

In this study, plasmid *RCO1*-pRS313-HA-SSN6 was cloned using PCR with Q5 polymerase (NEB; Ipswich, MA, USA) with primers TAL-187 and TAL-188 according to manufacture directions. The resulting PCR products were purified with QIAquick Gel Extraction Kit and subject to restriction digest with *Sac*I and *EcoR*I (NEB) and ligated into plasmid pRS313-3XHA-SSN6 using T4 ligase (NEB) according to manufacture directions, the resulting plasmid was sequence confirmed. Mutant plasmids for *RCO1* and *EAF3* were synthesized by Vector Builder (Chicago, IL, USA). All plasmids are listed in Supplementary Table S3.

Spotting Assays and Western Blotting.

In this assay, the *FLO8* gene (driven by the *GAL1* promoter) was modified to harbor an out-of-frame *URA3* gene containing a cryptic start site that becomes in frame and produces a

functional *URA3* transcript when the cryptic promoter within the gene is used. Plasmids were transformed into their respective cryptic reporter strains using the High Efficiency Yeast Transformation using Lithium Acetate/Single Stranded Carrier DNA/PEG method [8] with 500 ng of each plasmid. Selection for uptake of the plasmid was conducted on Synthetic Complete (SC) lacking Histidine (SC-His) agar plates. The Strains were grown in SC-His and subsequently used for spotting assay on selective plates, SC-His, SC-His-Ura+Glucose (Glu), and SC-His-Ura+Glactose (Gal) to look for cryptic transcription. Total protein was prepared as described [6] using 14 OD_{600} units of cell culture and SUTEB lysis buffer (10 mM Tris-HCl pH 8.0, 1% SDS, 8M Urea, 10 mM EDTA, and 0.01% bromophenol blue). The total protein samples (50 μL) were loaded on a 15% SDS PAGE gel and electrophoresed then subsequently transferred to PVDF membrane. For the Rco1 and Eaf3 westerns, the membrane was probed with aHA antibody (1:500, Proteintech: 51064-2-AP) as well as aG6PDH (1:10K, SigmaAldrich: A9521) (**Supplementary Fig. 8a-b**).

References

1 Zhang, W.-Z. *et al.* The protein complex crystallography beamline (BL19U1) at the Shanghai Synchrotron Radiation Facility. *Nuclear Science and Techniques* **30**, 170, doi:10.1007/s41365-019-0683-2 (2019).

2 Minor, W., Cymborowski, M., Otwinowski, Z. & Chruszcz, M. HKL-3000: the integration of data reduction and structure solution--from diffraction images to an initial model in minutes. *Acta Crystallogr D Biol Crystallogr* **62**, 859-866, doi:10.1107/S0907444906019949 (2006).

3 Otwinowski, Z. & Minor, W. Processing of X-ray diffraction data collected in oscillation mode. *Methods Enzymol* **276**, 307-326, doi:10.1016/S0076-6879(97)76066-X (1997).

4 Emsley, P. & Cowtan, K. Coot: model-building tools for molecular graphics. *Acta*

Crystallogr D Biol Crystallogr **60**, 2126-2132, doi:10.1107/S0907444904019158 (2004).

5 Adams, P. D. *et al.* PHENIX: building new software for automated crystallographic structure determination. *Acta Crystallogr D Biol Crystallogr* **58**, 1948-1954, doi:10.1107/s0907444902016657 (2002).

6 McDaniel, S. L. *et al.* Combinatorial Histone Readout by the Dual Plant Homeodomain (PHD) Fingers of Rco1 Mediates Rpd3S Chromatin Recruitment and the Maintenance of Transcriptional Fidelity. *Journal of Biological Chemistry* **291**, 14796-14802, doi:10.1074/jbc.M116.720193 (2016).

7 Keogh, M. C. *et al.* Cotranscriptional Set2 methylation of histone H3 lysine 36 recruits a repressive Rpd3 complex. *Cell* **123**, 593-605, doi:10.1016/j.cell.2005.10.025 (2005).

8 Gietz, R. D. & Woods, R. A. Transformation of yeast by lithium acetate/single-stranded carrier DNA/polyethylene glycol method. *Methods Enzymol* **350**, 87-96, doi:10.1016/s0076-6879(02)50957-5 (2002).

Supplementary Table S1. Data collection and refinement statistics

	Eaf3$^{218\text{-}401}$/Rco1$^{240\text{-}376}$/H3$^{1\text{-}12}$	Eaf3$^{218\text{-}401}$/Eaf7$^{108\text{-}143}$
**Data collection**		
Space group	C 1 2 1	C 1 2 1
Cell dimensions		
a, *b*, *c* (Å)	82.51, 75.55, 73.42	138.03, 42.09, 80.79
α, β, γ (°)	90, 114.19, 90	90, 95.35, 90
Resolution (Å)	33.49-1.60(1.63-1.60)	40.24-2.40(2.49-2.40)
R_{sym} or R_{merge}	0.037(0.485)	0.073(0.580)
$I / \sigma I$	24.6(2.3)	16.5(2.9)
CC1/2	0.999(0.826)	0.999(0.947)
Completeness (%)	99.6(94.4)	99.6(99.9)
Redundancy	6.2(3.9)	6.5(6.3)
**Refinement**		
Resolution (Å)	28.24-1.62	36.39-2.40
No. reflections	52126	18414
R_{work} / R_{free}	0.168/0.187	0.193/0.255
No. atoms		
Protein	2440	3181
Ligand/ion	2	N/A
Water	295	111
B-factors		
Protein	33.8	56.0
Ligand/ion	30.4	N/A
Water	38.9	58.1
R.m.s. deviations		
Bond lengths (Å)	0.012	0.008
Bond angles (°)	1.2	0.94
Ramachandran Plot favored/allowed/outliers (%)	98.0/2.0/0.0	99.2/0.8/0.0

*Values in parentheses are for highest-resolution shell.

Supplementary Table S2. Yeast strains used in this study.

Strain name	Description and genotypes	References
KLY443	FLO8-5' UTR (-170/-56)Δ::Nat.NT2-GAL1pr -HA3 FLO8-3' UTR (+2569/3619)D::URA3.Hph.NT1	(3)
KLY550	-3 UTR (+2569/3619)Δ::URA3.Hph.NT1 flo8p (-170/-56) Δ::Nat-NT2-TDH3p-HA3 rco1Δ::KanMX	(11)
YSM121	MATa, can1Δ::STE2pr-LEU2, lyp1Δ, flo8pΔ::Nat.NT2-GAL1p-HA3 flo8-3UTRΔ::URA3.Hph.MX6 eaf3Δ::KanMX	This study
yTAL-034	WT cryptic (KLY443) with empty plasmid	This study
yTAL-035	*RCO1*Δ cryptic (KLY550) with empty plasmid	This study
yTAL-036	*RCO1*Δ cryptic (KLY550) with WT Rco1-HA	This study
yTAL-037	*RCO1*Δ cryptic (KLY550) with mutant Rco1 D314-376-HA	This study
yTAL-038	*RCO1*Δ cryptic (KLY550) with mutant Rco1 F351D/L353D-HA	This study
yTAL-039	*EAF3*Δ cryptic (YSM121) with empty plasmid	This study
yTAL-040	*EAF3*Δ cryptic (YSM121) with WTEaf3-HA	This study
yTAL-041	*EAF3*Δ cryptic (YSM121) with mutant Eaf3 V233E/T240A-HA	This study
yTAL-042	*EAF3*Δ cryptic (YSM121) with mutant Eaf3 V233E/W236D-HA	This study
yTAL-043	*EAF3*Δ cryptic (YSM121) with mutant Eaf3 R300A/R303E-HA	This study

Supplementary Table S3. Plasmids and primers used in this study.

Plasmid name	Protein expressed	Plasmid backbone	Tag	Source	Ref
pRS313-3HA-SSN6	N/A	pRS313-3XHA-SSN6	N/A	Cloned in-house	(3)
RCO1-pRS313-HA-SSN6	Wild type Rco1-HA	pRS313-3XHA-SSN6	HA	Cloned in-house with Primer TAL-187 & TAL-188	This study (pTAL-129)
pRS313 *rco1* del314-376 HA	Mutant Rco1Δ314-376-HA	pRS313-3XHA-SSN6	HA	Vector Builder: VB220419-1423gjb	This study
pRS313 *rco1* F351D/L353D HA	Mutant Rco1 F351D/L353D-HA	pRS313-3XHA-SSN6	HA	Vector Builder: VB220419-1420xdh	This study
pRS313 *EAF3*-HA3-SSN6	Wild type Eaf3-HA	pRS313-3XHA-SSN6	HA	Cloned in-house	(3)
pRS313 *eaf3* V233E/T240A	Mutant Eaf3 V233E/T240A-HA	pRS313-3XHA-SSN6	HA	Vector Builder: VB220416-1031yrv	This study
pRS313 *eaf3* V233E/W236D	Mutant Eaf3 V233E/W236-HA	pRS313-3XHA-SSN6	HA	Vector Builder: VB220416-1030mcv	This study
pRS313 *eaf3* R300A/R303E	Mutant Eaf3 R300A/R303E-HA	pRS313-3XHA-SSN6	HA	Vector Builder: VB220416-1029mze	This study

Primer Name	Sequence (5'-3')	Restriction site [a]
TAL-187	GCTCACCGAGCTCGGCAACTTTCTTCCTCATGTAGG	*Sac*I
TAL-188	ACGCCGGAATTCTTCGGATTGGTTTCGGG	*EcoR*I
oSM128	CATCTGTGAGGCCTCGTCACTGGATTTACCCTATTGAAGAAC GTATAATGCGTACGCTGCAGGTCGAC	N/A
oSM129	GGAAGAACTAAATACTAGAAATAATCCCAAGCTAGAATATAA ACGTCTCAATCGATGAATTCGAGCTCG	N/A

[a] The restriction site sequences are underlined. N/A indicates not applicable.

References

3 Keogh, M. C. et al. Cell 123, 593-605 (2005).

11 McDaniel, S. L. et al. J. Biol. Chem. 291, 14796-14802 (2016).

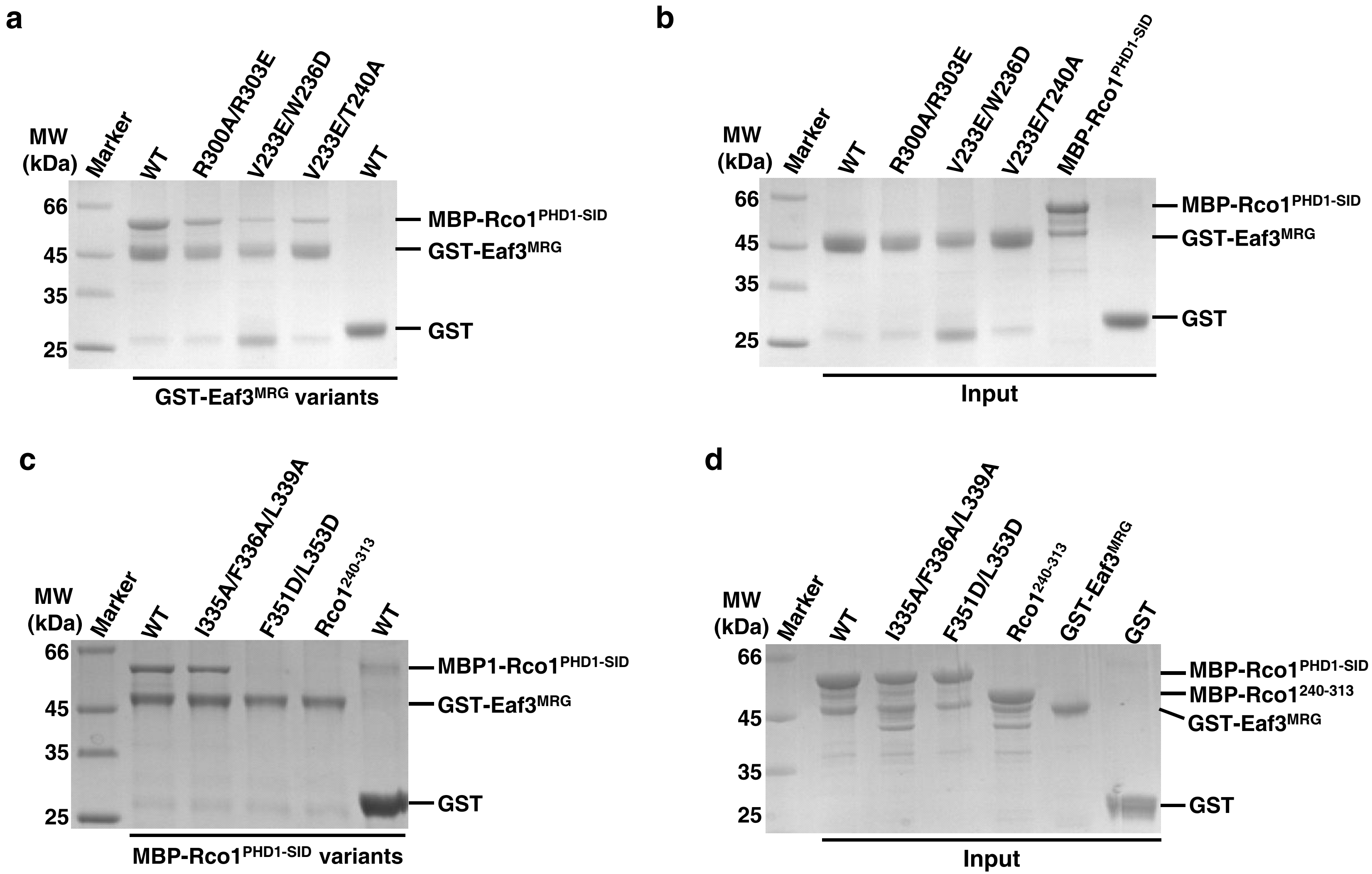

Supplementary Figure S1. Pull-down assay for Eaf3^{MRG} binding to Rco1$^{PHD1-SID}$. **a** GST pull-down assay for GST-Eaf3^{MRG} variants binding to MBP-Rco1$^{PHD1-SID}$ with GST as the control. **b** Inputs for wild type GST-Eaf3^{MRG} and its mutants (lanes 2-5), MBP-Rco1$^{PHD1-SID}$ (lane 6), and GST (lane 7). **c** GST pull-down assay for GST-Eaf3^{MRG} binding to MBP-Rco1$^{PHD1-SID}$ variants with GST as the control. **d** Inputs for MBP-Rco1$^{PHD1-SID}$ and its mutants (lanes 2-4), truncated form of Rco1$^{PHD1-SID}$ (Rco1$^{240-313}$, lane 5), GST-Eaf3^{MRG} (lane 6) and GST (lane 7).

Supplementary Figure S1

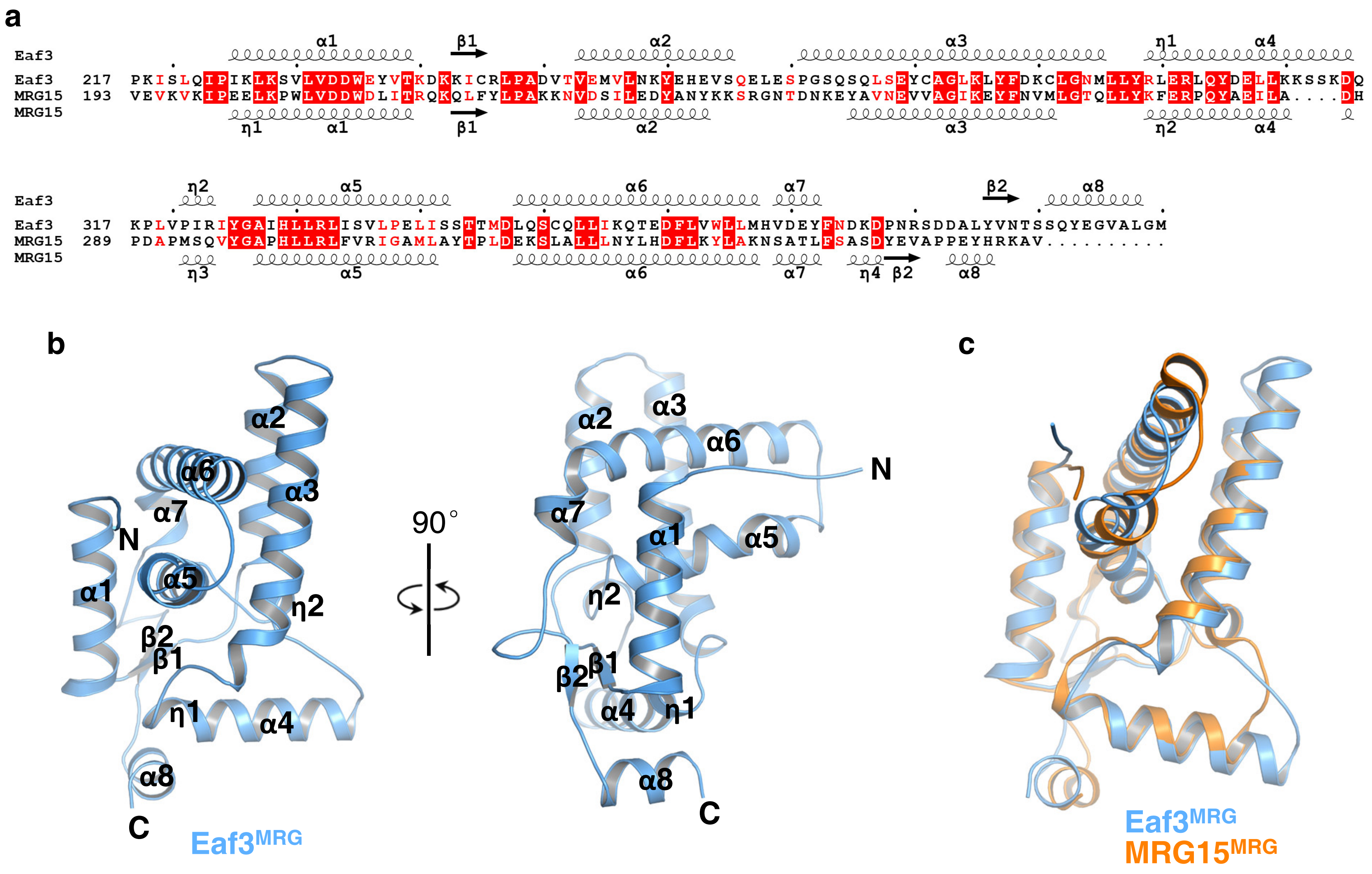

Supplementary Figure S2. Structure of Eaf3^{MRG}. **a** Sequence alignment of *S. cerevisiae* Eaf3 (NP_015348.1) and human MRG15 (NP_001252532.1). The secondary structures of Eaf3 and MRG15 are shown at the top and bottom of sequences, respectively. **b** The three-dimensional structure of Eaf3^{MRG}. **c** Superimposition of the structures of Eaf3^{MRG} and MRG15^{MRG} (PDB: 2AQL).

Supplementary Figure S2

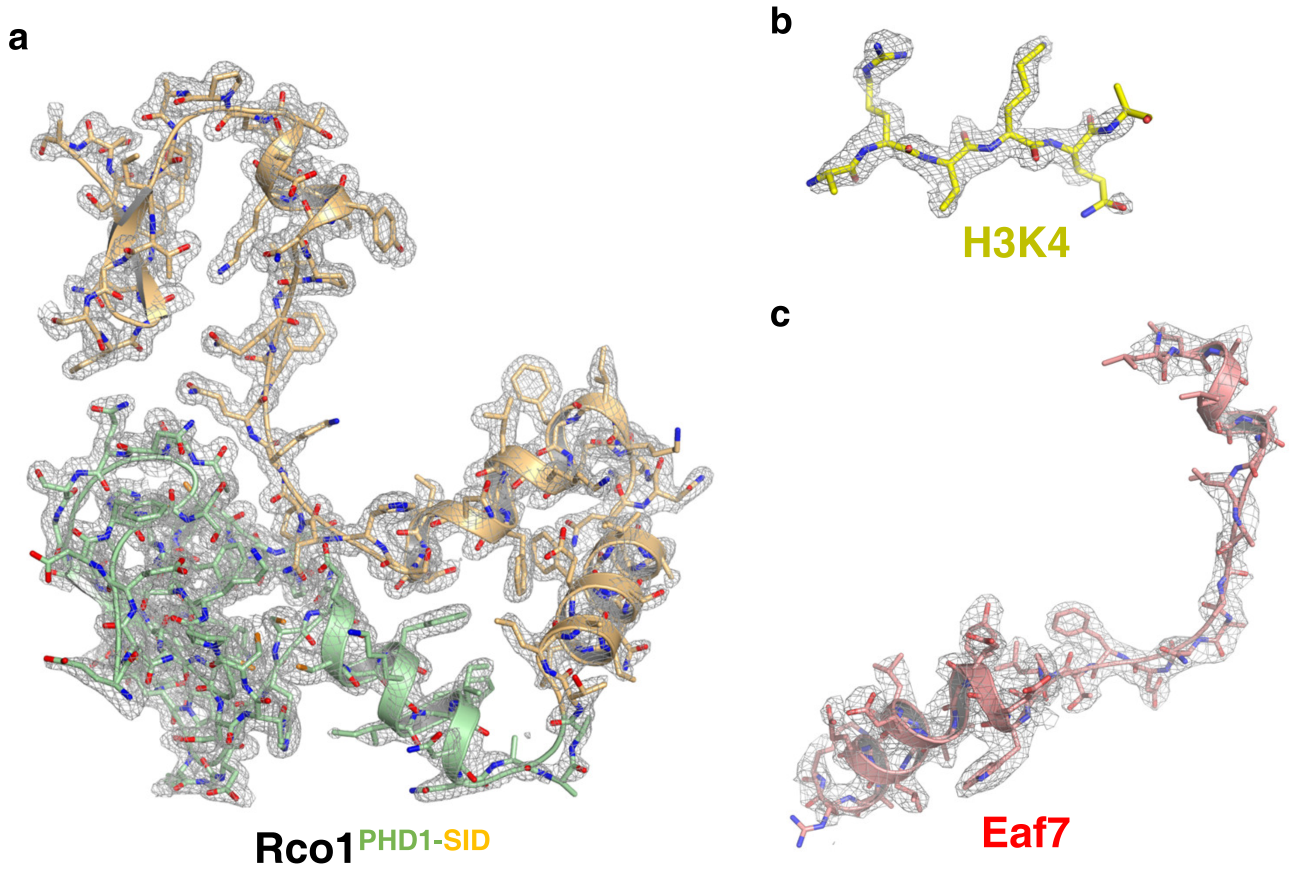

Supplementary Figure S3. The 2|Fo|-|Fc| maps of **a** Rco1$^{PHD1\text{-}SID}$, **b** unmodified H3K4, and **c** Eaf7 are shown at 1.0 σ level to show the quality of solved crystal structures.

Supplementary Figure S3

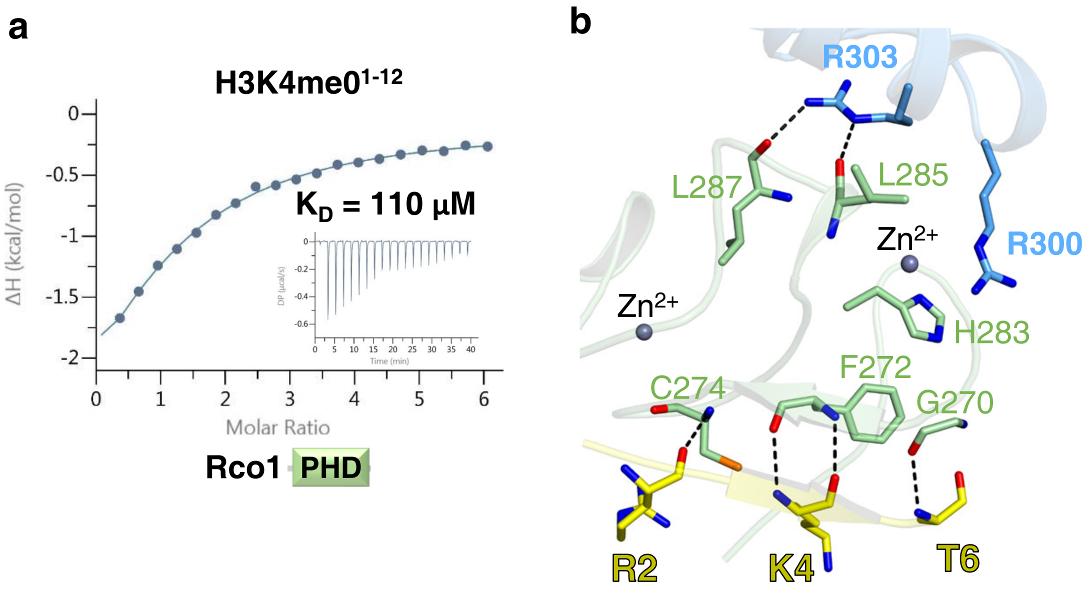

Supplementary Figure S4. Eaf3^{MRG} reinforces the Rco1^{PHD1}-H3K4me0 binding allosterically. **a** ITC binding curves for Rco1^{PHD1} with H3K4me0. **b** Allosteric regulatory role of Eaf3^{MRG}. Arg300 and Arg303 of Eaf3^{MRG}, and Arg2, Lys4, and Thr6 of H3K4me0 are shown in blue and yellow sticks, respectively. Rco1^{PHD1} residues involved in the interactions with Eaf3^{MRG} and H3K4me0 are shown in green sticks. The Zn ions are shown in grey sphere.

Supplementary Figure S4

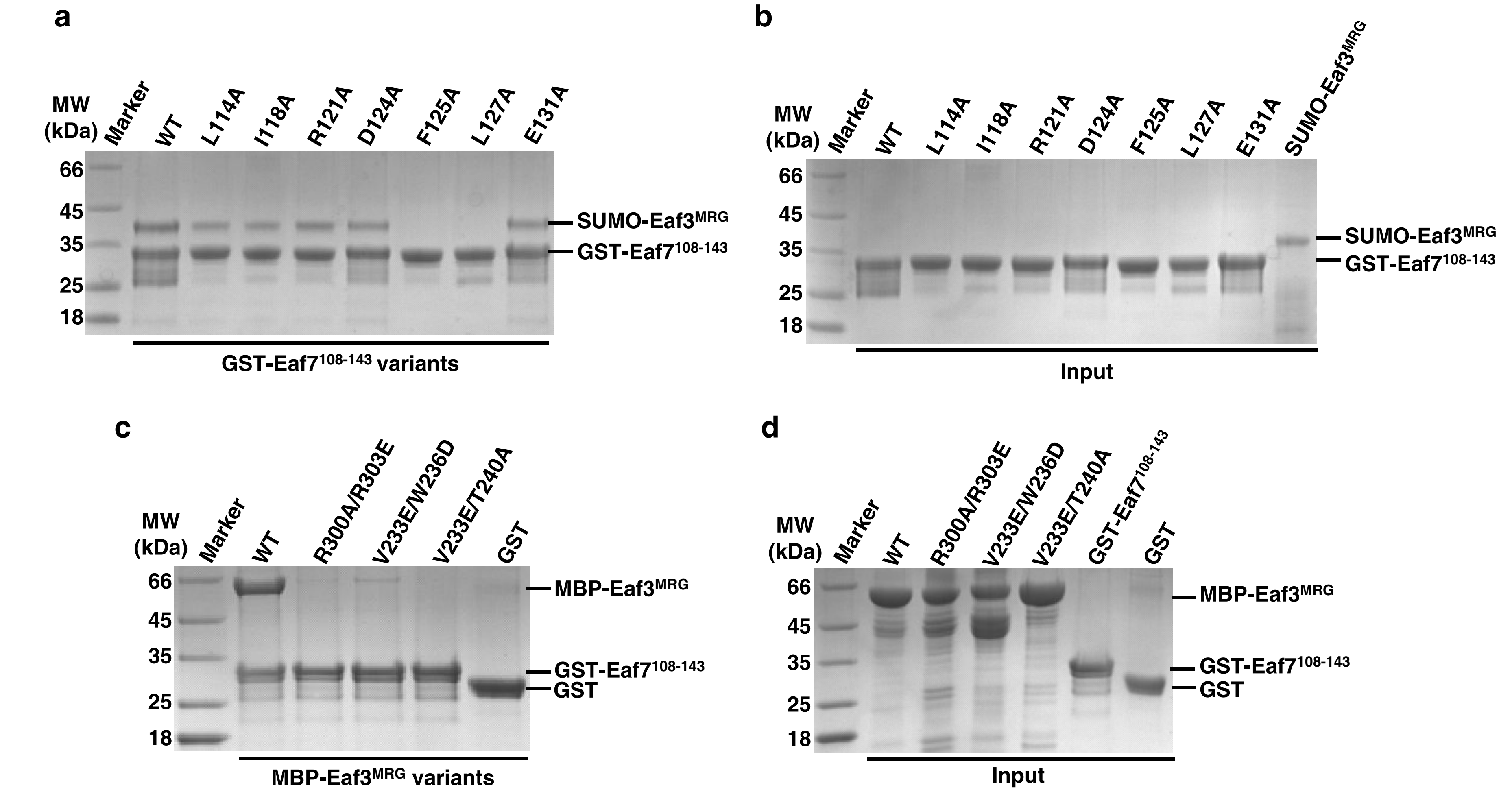

Supplementary Figure S5. Pull-down assay for Eaf7$^{108\text{-}143}$ binding to Eaf3^{MRG}. **a** GST pull-down assay for GST-Eaf7$^{108\text{-}143}$ variants binding to SUMO-Eaf3^{MRG}. **b** Inputs for wild type GST-Eaf7$^{108\text{-}143}$ and its mutants (lanes 2-9), and SUMO-Eaf3^{MRG} (lane 10). **c** GST pull-down assay for GST-Eaf7$^{108\text{-}143}$ binding to MBP-Eaf3^{MRG} variants with GST as the control. **d** Inputs for wild type MBP-Eaf3^{MRG} and its mutants (lanes 2-5), GST-Eaf7$^{108\text{-}143}$ (lane 6) and GST (lane 7).

Supplementary Figure S5

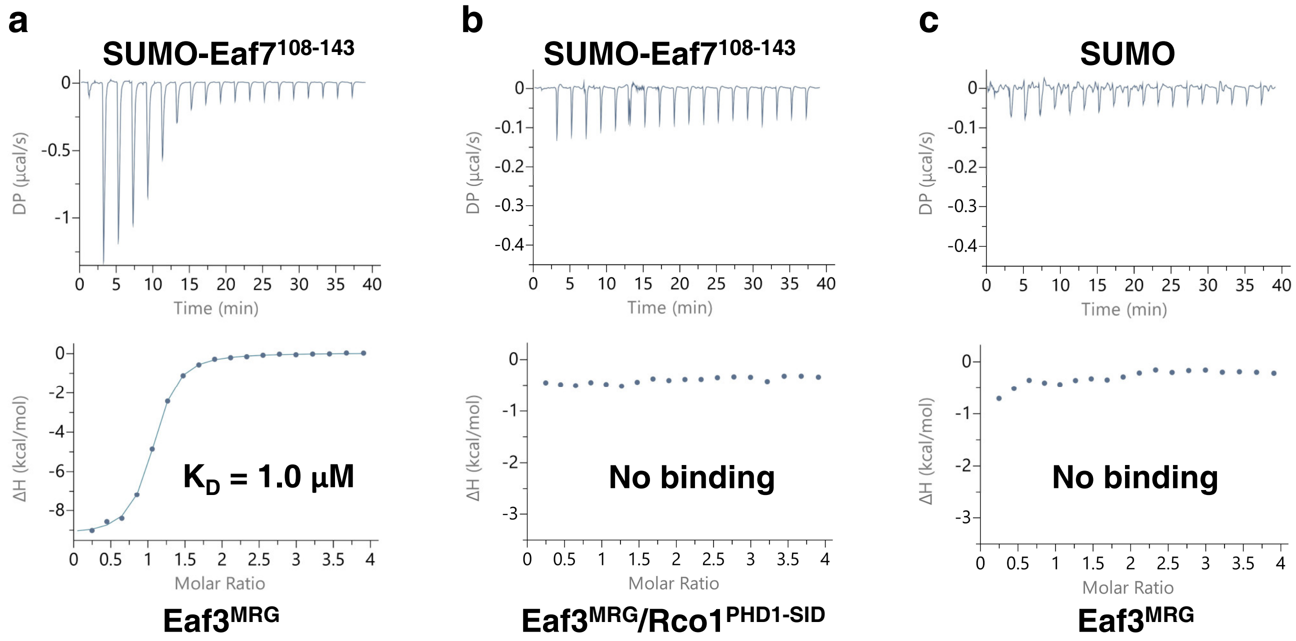

Supplementary Figure S6. a ITC curve for SUMO-Eaf7$^{108\text{-}143}$ binding to Eaf3^{MRG}. **b** ITC curve for SUMO-Eaf7$^{108\text{-}143}$ binding to the Eaf3^{MRG}/Rco1$^{PHD1\text{-}SID}$ complex. **c** ITC curve for SUMO binding to Eaf3^{MRG} .

Supplementary Figure S6

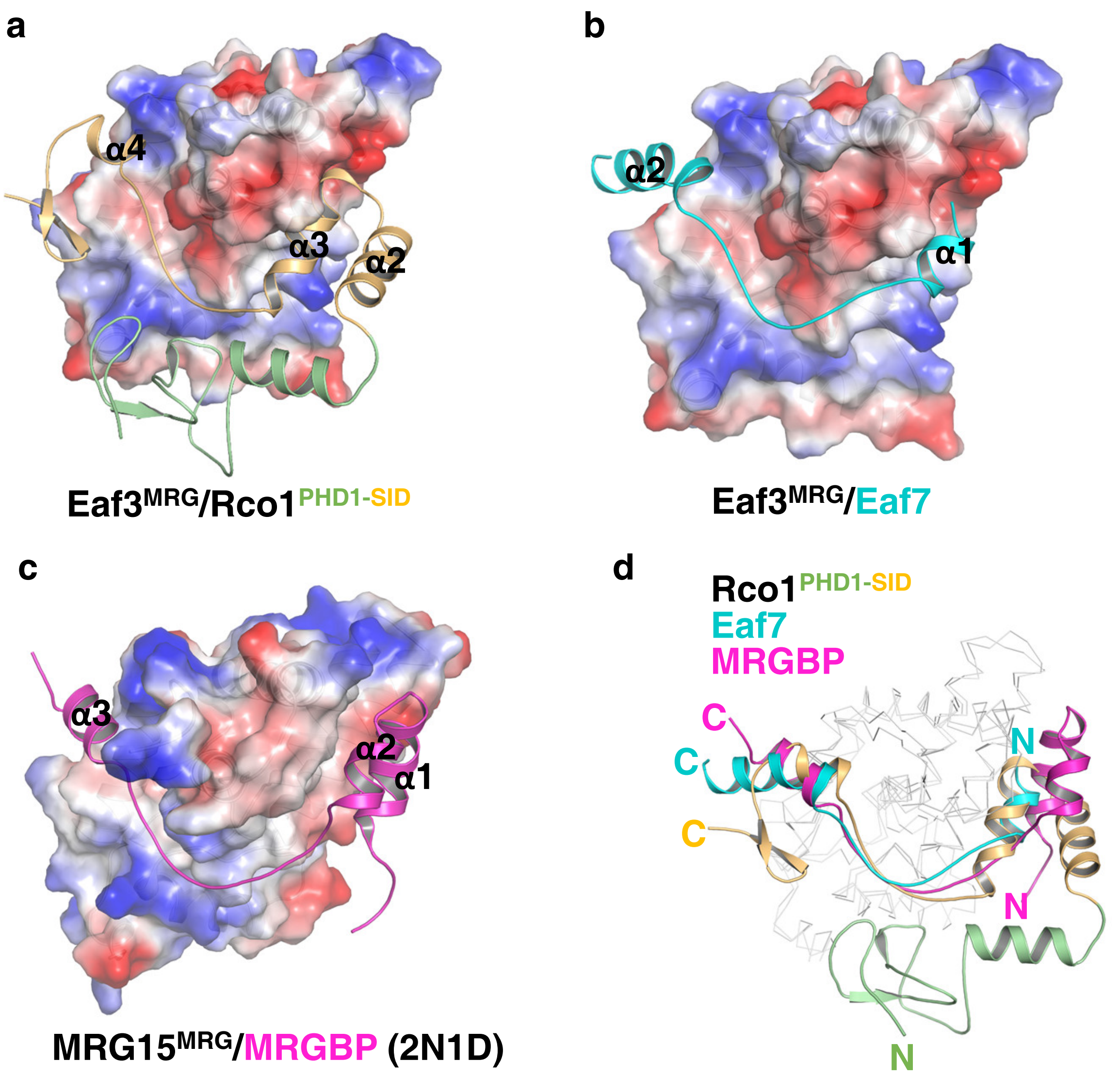

Supplementary Figure S7. Comparing the ligand binding modes of MRG domains. **a** Electrostatic surface of Eaf3^{MRG} bound with Rco1$^{PHD1\text{-}SID}$ with Rco1$^{PHD1\text{-}SID}$ shown in the same way as in Figure 1f. **b** Electrostatic surface of Eaf3^{MRG} bound with Eaf7$^{108\text{-}143}$ with Rco1$^{PHD1\text{-}SID}$ shown in cyan cartoon. **c** Electrostatic surface of MRG15^{MRG} bound with a fragment of MRGBP (PDB: 2N1D) with MRGBP shown in magenta cartoon. **d** All ligand-bound MRG structures are superimposed on the Eaf3^{MRG}. The ligands are colored the same as shown in Supplementary Figure S5a-S5c.

Supplementary Figure S7

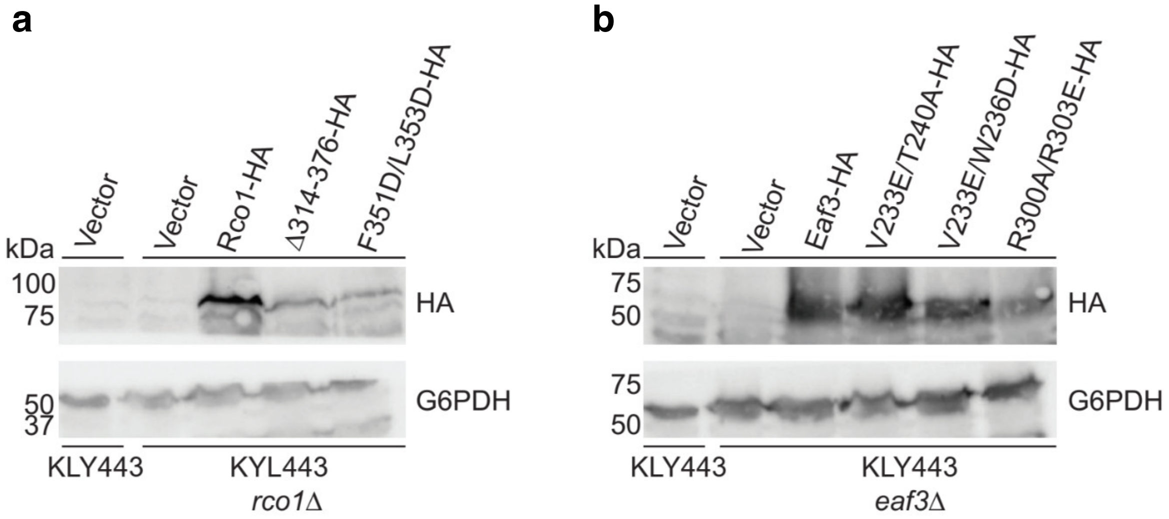

Supplementary Figure S8. a-b Western blot images of the HA-tagged Eaf3 or Rco1in their respective strains. G6PDH was used as a loading control.

Supplementary Figure S8
